# Therapeutic Effects of Plant Extracts of *Anoectochilus roxburghii* on Side Effects of Chemotherapy in BALB/c Breast Cancer Mice

**DOI:** 10.3390/plants12132494

**Published:** 2023-06-29

**Authors:** Chi-Feng Cheng, Chen-Wen Lu, Wen-Jhen Wu, Li-Yu Su, Thi Kim Ngan Nguyen, Szu-Chuan Shen, Chia-Ying Lien, Wu-Chang Chuang, Ming-Chung Lee, Chung-Hsin Wu

**Affiliations:** 1 School of Life Science, National Taiwan Normal University, Taipei 106, Taiwan; draculachi@yahoo.com.tw (C.-F.C.); kumo.lu@gmail.com (C.-W.L.); efgy78@gmail.com (W.-J.W.); marynguyen@ntnu.edu.tw (T.K.N.N.); scs@ntnu.edu.tw (S.-C.S.); 2 Department of Oncology, Taipei City United Hospital, Renai Branch, Taipei 106, Taiwan; 3 Department of Physiology, College of Medicine, National Taiwan University, Taipei 100, Taiwan; julia10025@gmail.com; 4 Master Program of Sport Facility Management and Health Promotion, National Taiwan University, Taipei 106, Taiwan; chiayinglien@ntu.edu.tw; 5 Sun Ten Pharmaceutical Co. Ltd., New Taipei City 231, Taiwan; cwctd331@sunten.com.tw; 6 Brion Research Institute of Taiwan, New Taipei City 231, Taiwan; mileslee@sunten.com.tw

**Keywords:** chemotherapy reliever, breast cancer, doxorubicin, cardiotoxicity, myelosuppression, immunodeficiency, osteoporosis, mice

## Abstract

Breast cancer is the most common cancer in women, and chemotherapy is an effective treatment. However, chemotherapy often causes adverse side effects such as cardiotoxicity, myelosuppression, immunodeficiency, and osteoporosis. Our study focused on the alleviating effects of *Anoectochilus roxburghii* extracts (AREs) on the adverse side effects of chemotherapy in mice with breast cancer. We individually evaluated the antioxidant capacity and cytotoxicity of the AREs using DPPH and MTT assays. We also examined the effects of the AREs on intracellular F-actin, reactive oxygen species (ROS), and the mitochondrial membrane potential (MMP) of 4T1 cancer cells before and after doxorubicin (DOX) treatment. Our results showed that ARE treatment enhanced the effects of DOX chemotherapy by promoting cell morphology damage, oxidative stress, and ROS generation, as well as by reducing MMP in the 4T1 breast cancer cells. By using BALB/c mice with breast cancer with DOX treatment, our results showed that the DOX treatment reduced body weight, blood pressure, and heart rate and induced myelosuppression, immunodeficiency, cardiotoxicity, and osteoporosis. After oral ARE treatment of BALB/c mice with breast cancer, the chemotherapeutic effects of DOX were enhanced, and the adverse side effects of DOX chemotherapy were alleviated. Based on the above results, we suggest that AREs can be used as an adjuvant reliever to DOX chemotherapy in BALB/c mice with breast cancer.

## 1. Introduction

Breast cancer is the most common cancer in women, and chemotherapy is the medical method for breast cancer treatment. It can be used as an adjuvant therapy for early breast cancer and metastatic breast cancer [1]. Many adverse side effects, such as weight loss, myocardial injury, bone marrow hematopoietic defects, immunocompromised function, and osteoporosis, often occur when patients receive moderate to highly toxic chemotherapy [2]. Among many chemotherapeutic drugs, doxorubicin (DOX) is an effective chemotherapeutic drug that is commonly used to treat human breast cancer [2]. However, DOX may cause adverse side effects, such as cardiotoxicity, myelosuppression, immunodeficiency, and osteoporosis [3]. Clinical evidence suggests that DOX induces severe osteoporosis and osteoarthritis in patients with cancer undergoing chemotherapy [4]. Finding alternative therapies that can reduce the adverse effects of cancer chemotherapy should be our goal.

To explore how to reduce the side effects of cancer chemotherapy, we have published several journal papers on the protective effect of traditional Chinese herbal medicines on the adverse side effects of cancer chemotherapy. For example, our previous study revealed that DOX-treated mice exhibited myocardial oxidative stress, inflammation, and apoptosis. In contrast, the herbal formula *Sheng Mai San* plus *Danshen* provided cardioprotection in DOX-treated mice by suppressing oxidative stress, inflammation, and apoptosis [5]. Another study of ours showed that treatment with the herbal formula *Guilu Erxian Glue* could alleviate chemotherapy-related bone loss [6]. Our recent findings revealed that extracts of the herbal formula *Cordyceps militaris* can enhance antioxidant-stress-related SOD2 protein expression but inhibit inflammation-related TNF-α protein expression in the myocardial tissue of chemotherapy-treated mice [7]. In addition, *Cordyceps militaris* can enhance IgG and IgA expressions but inhibit IgE expression in the serum of chemotherapy-treated mice [7]. As suggested by these studies, we suggest that traditional Chinese herbal medicines are worth developing as candidates for alleviating the adverse side effects of chemotherapy.

Among the many Chinese herbal medicines we reviewed, *Anoectochilus roxburghii* (Wall.) Lindl. (AR) was shown to have the ability to alleviate oxidative stress and inflammation [8]. Phytochemical investigations have revealed that AR is rich in various functional components, such as kinsenoside, polysaccharides, flavonoids, glycosides, organic acids, steroids, triterpenes, and alkaloids [9,10]. AR has many pharmacological activities, including antidiabetic [11], blood vessel protection [12], liver protection [13], antioxidant [14], antibacterial [15], and anticancer effects [16]. In recent years, AR has been widely used in medicine, functional foods, beauty, and other fields and is considered one of the most valuable medicinal plants. AR may be a candidate for alleviating the adverse side effects of chemotherapy. Kinsenoside, 4R-4-O-(β-D- glucopyranosyl)-2(5H)-furanone, is a major bioactive component derived from the whole plants of the genus *Anoectochilus*. Moreover, it has a broad range of pharmacological functions and has been shown to have many pharmacological effects, such as liver protection, anti-inflammation, blood vessel protection, and anti-osteoporosis [17]. Polysaccharides are a main component of AR. More than 30 different polysaccharide structures have been extracted from the whole plants, leaves, and roots of A. roxburghii. AR polysaccharides are heteropolysaccharides with different proportions of nine types of monosaccharides, glucose, galactose, arabinose, xylose, rhamnose, ribose, fucoid sugar, galacturonic acid, and glucuronic acid [9]. AR polysaccharides can exert a wide variety of remarkable physiological functions such as antidiabetic, hepatoprotective, antioxidant, anti-inflammatory, antitumor, immunomodulatory, sedative, hypnotic, metabolic modulation, anti-aging, and hypolipidemic activities [18,19]. Cancer patients receiving chemotherapy are often prone to infection, leading to complications such as inflammation, bleeding, sepsis, and diarrhea [20]. A recent review paper suggested that chemotherapy-related inflammation and complications can be reduced or avoided by restoring the gut microbiota [21]. AR polysaccharides can promote intestinal health by changing the intestinal microbiota [22].

We believe that AREs have the medical potential to be developed as an adjuvant reliever for alleviating chemotherapy-induced adverse side effects. Therefore, our study focused on the alleviating effects of AREs on the adverse side effects of chemotherapy using BALB/c mice with breast cancer.

## 2. Materials and Methods

### 2.1. Preparation of the AREs

**Figure 1:**
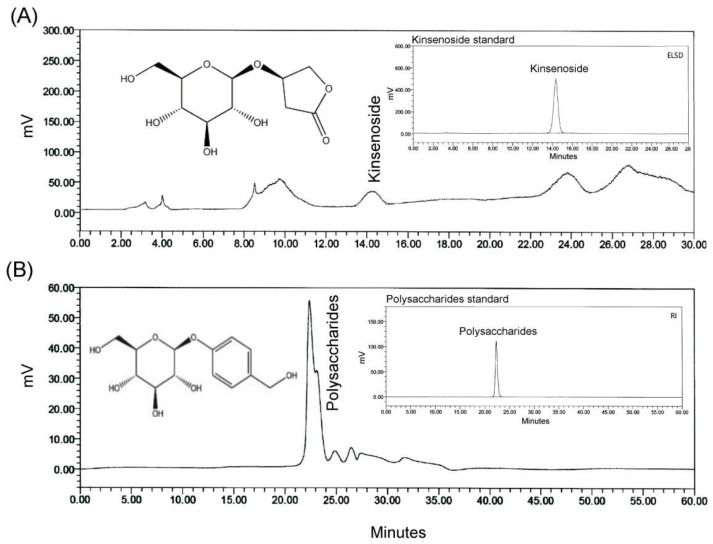
The HPLC fingerprint of the AREs included (**A**) AR kinsenoside (ARK) and (**B**) AR polysaccharides (ARPs). The chemical structures of ARK and ARPs are shown in the upper left blank space. The standard chromatographs of ARK and ARPs are shown in the upper right box. AREs: *Anoectochilus roxburghii* extracts.

In this study, AREs were selected as an adjuvant reliever for alleviating chemotherapy-induced adverse side effects. The AREs were purchased from Sun Ten Pharmaceutical Company (New Taipei City, Taiwan). The AREs were in the form of powder, which was mixed evenly with distilled water. In order to determine the adequate intake of AREs required by each mouse, the experimental treatment was fed via tube feeding at a dose of 150 mg/kg. The analysis of the main active compounds of the AREs was carried out by HERBIOTEK Company (New Taipei City, Taiwan). All compounds of the AREs were dissolved in distilled water/methanol using high-performance liquid chromatography (HPLC) for chromatographic fingerprinting. The active compounds of the AREs were deduced by comparing the retention times of individual peaks with those of the actual control. After the AREs were extracted with methanol, they were partitioned with different polar solvents of n-hexane, ethyl acetate, n-butanol, and water to obtain the highest proportion of the aqueous extract. Figure 1 shows the HPLC fingerprint of the AREs, which included kinsenoside and polysaccharides.

### 2.2. DPPH Assay of the AREs

In this experiment, a DPPH (α, α-diphenyl-β-pricrylhydrazyl) anti-free radical test was used to detect the antioxidant capacity of the AREs. The AREs were diluted with 100 μL of 1.5 mM/mL DPPH (D9132, Sigma–Aldrich Co., St. Louis, MO, USA) and evenly mixed in a 96-well plate; then, the absorbance was recorded after the samples were allowed to stand at room temperature for 30 min. We recorded color changes at a wavelength of 517 nm using a microplate spectrophotometer (µQuant, Biotek Intruments, Inc., Winowski, VT, USA). Appropriate blank treatments and standards (L-ascorbic acid; A5960, Sigma–Aldrich Co.) were also recorded. The inhibitory activity (%) of the DPPH clearance of the AREs was calculated using the following formula: DPPH clearance (%) of AREs = 100 × [(ARE + DPPH absorbance) − (ARE blank absorbance)]/[(DPPH absorbance) − (methanol absorbance)].

### 2.3. MTT Assay of 4T1 Breast Cancer Cells

In this experiment, 3-(4,5-Dimethylthiazol-2-yl)-2,5-diphenyltetrazolium bromide (MTT, M5655, Sigma–Aldrich Co.) was used to examine the protective effect of the AREs on the injury of 4T1 breast cancer cells induced by DOX. The 4T1 breast cancer cells were purchased from the Bioresource Collection and Research Center (Hsinchu, Taiwan). The 4T1 breast cancer cells were cultured in RPMI1640 medium supplemented with 10% heat-inactivated FBS, 100 unit/mL penicillin, 100 μg/mL streptomycin, and 2 mmol/L glutamine. All 4T1 breast cancer cells were incubated at 37 °C with 5% CO_2_ for 24 h under normoxia conditions. In this study, we used 10 µg/mL DOX (D1515, Sigma–Aldrich Co.) as the dose for the chemotherapy-damaged 4T1 breast cancer cells. According to the results of Bao et al. [23], the treatment of 4T1 breast cancer cells with 0.17 mM DOX induced 50% growth inhibition (IC_50_) within 48 h. Their result was very similar to the result of Du et al. [24]. Here, we based this experiment on the study of Kim et al. [25], which used 10 μg/mL DOX to treat tumor cells. A total of 5 × 10^5^ cells/mL 4T1 breast cancer cells was added to a 24-well plate, 10 μg/mL DOX (D1515, Sigma–Aldrich Co.) was partially added, and 0, 1, 5, 10, 25, and 50 µg/mL of ARE samples were added to the 4T1 breast cancer cells, followed by culturing within 24 h. After adding 0.5 mg/mL of MTT solution (M5655, Sigma–Aldrich Co., St. Louis, MO, USA) and culturing for 2 h, the supernatant was removed, and 100 μL/well of DMSO organic solvent was added, followed by 5 min of shaking and then absorbance measurement at 570 nm.

### 2.4. Immunofluorescence and Flow Cytometry Assay of 4T1 Breast Cancer Cells

In this study, Rhodamine Phalloidin dye R-415 (Thermo Fisher Scientific, Waltham, MA, USA) was used for cytoskeleton labeling, and 4′,6-Diamidino-2- Phenylindole, Dihydrochloride (DAPI) dye (Thermo Fisher Scientific), was used for nuclei labeling in the 4T1 breast cancer cells. The prepared 4T1 breast cancer cell specimens were securely mounted with coverslips. Fluorescence images of the 4T1 breast cancer cells were viewed using a Leica DM IRB inverted fluorescence microscope and analyzed using Leica Application Suite software (LAS) V4.12 (Wetzlar, Germany). To visualize the cytoskeleton labeled by Rhodamine, the cells were irradiated at 540 nm, and the dye emission was detected at 565 nm. The blue fluorescence of DAPI-labeled nuclei was excited at 358 nm, and the emission was detected at 461 nm. In addition, dichlorofluorescein diacetate (DCFDA) (Molecular Probes, Eugene, OR, USA) was used for reactive oxygen species (ROS) labeling, and the 3,3’-dihexyloxa- carbocyanine iodide (DiOC6 (3)) method (Sigma–Aldrich Co.) was used for mitochondrial membrane potential labeling in the 4T1 breast cancer cells. The mean fluorescence intensity (FL-1) of the 4T1 breast cancer cells was analyzed using an FACS Calibur flow cytometer (Becton–Dickison, PharMingen, Lake Franklin, NJ, USA).

### 2.5. BALB/c Mice Preparation

This experiment used BALB/c mice, which were purchased from the BioLASCO Breeding Center of Lesco Biotechnology Co., Ltd. (Yilan, Taiwan), and the animal experiments were approved by the Animal Care and Use Supervision Committee of National Taiwan Normal University (Permit number: NTNU/Animal Use/No. 109007). All BALB/c mice were housed in animal rooms at 22 °C ± 2 °C with a 12 h light/dark cycle, and animals had ad libitum access to water and food. Animal experiments were performed in accordance with the International Guidelines for the Care and Use of Laboratory Animals, and the animal experiments were considered to comply with the 3R principles (replacement, reduction, and improvement) to optimize the experimental design.

A total of 32 BALB/c mice born at 20 weeks were selected, implanted subcutaneously with 5 × 10^6^ 4T1 breast cancer cells, and then divided into the Sham treatment group (Sham group, N = 8), ARE treatment group (ARE group, N = 8), DOX chemotherapy treatment group (DOX group, N = 8), and DOX chemotherapy after oral ARE treatment group (ARE+DOX group, N = 8).

The animal experiment was conducted over a total of four weeks. We measured the body weight, blood pressure, and heart rate of the mice at a fixed time before sacrifice. The ARE and ARE+DOX groups were orally treated with AREs (150 mg/kg), and the Sham and DOX groups were fed ad libitum every morning and evening from the first to the third week. During the third week, the DOX and ARE+DOX groups were administered three intraperitoneal injections of DOX (2.4 mg/kg), and the Sham and ARE groups were administered three intraperitoneal injections of saline solution every other day. In the fourth week, mice were sacrificed after anesthesia, and then the blood, myocardium, tibia, and tumor tissues were collected for further analysis.

### 2.6. Cardiac Function Measurement in BALB/c Mice

We evaluated the cardiac function of the BALB/c mice via color Doppler M-mode echocardiography (S-Sharp Corporation, Taipei, Taiwan). After anesthetizing the BALB/c mice with 2% isoflurane gas (Baxter Healthcare, New Providence, RI, USA), they were placed on a heated work platform to monitor ECG and respiratory gating. Changes in heart rate and cardiac output were measured and compared between the Sham and AR- and DOX-treated BALB/c mice using M-mode and color Doppler images. The average value of the three stable consecutive cardiac cycles was taken for each mouse.

### 2.7. Hematological and Serum Immunoglobulin Analysis in BALB/c Mice

Blood samples were collected from the veins after the BALB/c mice were anesthetized. For hematology analysis, blood samples were collected, and a small drop of blood was dropped on one side of a slide about 1 cm away from the edge; then, the blood was evenly spread on the other side, and the blood smear was left to dry naturally for later use. Then, we placed the prepared dry smear on a staining tank and completely covered the slide with Liu’s A solution for 30 s. Afterwards, Liu’s B solution and Liu’s A solution were added and fully mixed, and the slide was covered. After the stained blood smear was dried in the shade, we placed the slide in a microscope for direct observation.

For serum immunoglobulin analysis, the blood samples were placed in heparinized blood collection tubes (Terumo Inc., Tokyo, Japan); then, the serum samples were analyzed using the mouse Immunoglobulin A (mIgA) Enzyme-Linked Immunosorbent (ELISA) Kit (Abcam, Cambridge, MA, USA) and QuickDetect^TM^ Immunoglobulin E (IgE), Immunoglobulin G (IgG) ELISA Kit (BioVision, Milpitas, CA, USA). All ELISA methods were performed according to the manufacturers’ instructions. After adding the stop solution, the absorbance value (OD) was read at 450 nm using a Multiskan™ GO microplate spectrophotometer reader (Thermo Scientific™, Waltham, MA, USA).

### 2.8. IHC Staining Analysis of Myocardial Tissue in BALB/c Mice

The BALB/c mice were anesthetized and perfused with PBS containing 4% formaldehyde (EM-grade glutaraldehyde solution, Sigma–Aldrich Co.). Heart tissue samples from the BALB/c mice were fixed with 4% formaldehyde (Sigma–Aldrich Co.) and embedded in paraffin. Tissue specimens were cut into sections that were 5 μm thick using a tissue microtome, and then the sections were mounted on glass slides. Some myocardial tissue sections were stained with hematoxylin and eosin (H&E) (Sigma–Aldrich Co.) to assess tissue integrity. Other myocardial tissue sections were subjected to immunohistochemical (IHC) staining with SOD2 (Cat. numbers ab110300; Abcam) and purified rat antimouse tumor necrosis factor (TNF)-α (Cat. numbers #3707; Cell Signaling Technology, Danvers, MA, USA) for 1 h at room temperature. Incubation with biotinylated secondary antibody (NovolinkTM Polymer Detection System l, Leica Biosystems Newcastle Ltd., Newcastle, UK) for 30 min and avidin–biotin–horseradish peroxidase (HRP) complex (Novolink™ Polymer Detection System l, Leica Biosystems Newcastle Ltd.) for an additional 30 min was carried out. Immunostaining was visualized using DAB Chromogen (NovolinkTM Polymer Detection System 1, Leica Biosystems Newcastle Ltd.), and slides were counterstained with hematoxylin (NovolinkTM Polymer Detection System 1, Leica Biosystems Newcastle Ltd.).

### 2.9. Computed Tomography of the Tibia in BALB/c Mice

We evaluated the bone mineral density in the tibia of all BALB/c mice using computed tomography measurements that were performed by the Taiwan Mouse Clinic using a Skyscan 1076 micro-CT device (Skyscan, Aartselaar, Belgium). The scanning parameters were set as follows: 49 kV, 200 mA, 500 ms, and a voxel resolution of 18.27 mm. The microtomographic images were imported into CTAn V.1.18 software (Skyscan), and the tibial bone density and trabecular porosity size were calculated using Image J (Rasband, W.S., ImageJ, U.S. National Institutes of Health, Bethesda, MD, USA).

### 2.10. Statistical Analysis

In this study, SigmaPlot 12.5 (Systat Software Inc, Chicago, IL, USA) was used for data analysis and chart production. The data are presented as the mean ± standard error of the mean (SEM). Differences among the different groups of BALB/c mice were assessed using one-way or two-way analysis of variance (ANOVA). The Student–Newman–Keuls multiple comparisons post hoc test was performed when a significant F-value was obtained. Significance was defined as *p* < 0.05.

## 3. Results

### 3.1. Antioxidant Capacity and Cytotoxicity of AREs

**Figure 2:**
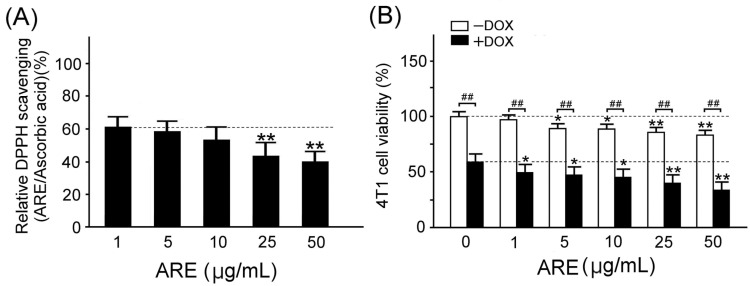
Antioxidant capacity and cytotoxicity of ARE treatments: (**A**) The quantified DPPH free radical scavenging activities of the ARE treatments relative to the standard (ascorbic acid) were significantly decreased with the concentrations of the ARE treatments (N = 3 for each group, ** *p* < 0.01, one-way ANOVA followed by the Student–Newman–Keuls multiple comparisons post hoc test). (**B**) The quantified 4T1 breast cancer cell viability obtained via the MTT assay was significantly reduced after DOX (10 µg/mL) treatment, and the ARE treatments (1–50 µg/mL) further decreased the cell viability of 4T1 breast cancer cells. Data are shown as the mean ± SEM (N = 3 for each group, ** *p* < 0.01, * *p* < 0.05, ^##^ *p* < 0.01, two-way ANOVA followed by the Student–Newman– Keuls multiple comparisons post hoc test).

Through the DPPH (1,1-diphenyl-2-picrylhydrazyl) assay, our results showed that the free radical scavenging ability of the ARE treatment was gradually decreased with the treated dose. Figure 2A shows the quantified DPPH free radical scavenging activities of the ARE treatments relative to the standard (L-ascorbic acid). We found that the reducing power of the DPPH scavenging values showed a good linear relationship in both the standard used and AREs (R^2^ = 0.975), and that the relative free radical scavenging activities were significantly decreased with ARE treatment concentrations of 25 and 50 µg/mL (*p* < 0.01). This result reveals that AREs under lower-dose treatments have a better antioxidant capacity to scavenge free radical damage than those under higher-dose treatments.

Through the MTT (3-(4,5-Dimethylthiazol-2-yl)-2,5- diphenyltetrazolium bromide) assay, we examined the effects of the AREs on the cell viability of 4T1 breast cancer cells with and without DOX treatment. Our results showed that DOX treatment caused chemotherapy damage in the 4T1 breast cancer cells because it significantly reduced cell viability (*p* < 0.01, Figure 2B). ARE treatment significantly enhanced the DOX-induced cell viability reduction in the 4T1 breast cancer cells (*p* < 0.01–0.05, Figure 2B). Figure 2B reveals that the ARE treatments were effective in potentiating DOX-induced chemotherapeutic damage in the 4T1 breast cancer cells.

### 3.2. Synergistic Damage of ARE and DOX Treatments in 4T1 Breast Cancer Cells

**Figure 3:**
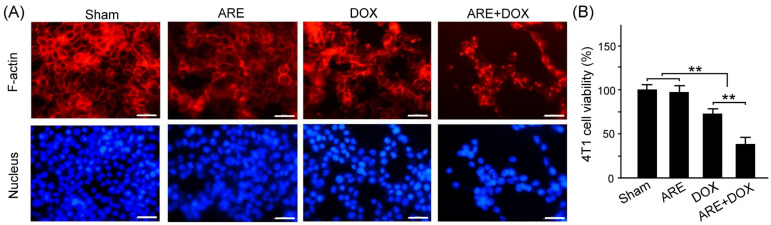
Effect of ARE treatment on the morphology of F-actin structures in 4T1 breast cancer cells with DOX chemotherapy: (**A**) Rhodamine Phalloidin and DAPI (blue) staining of 4T1 breast cancer cells untreated and treated with DOX (10 µg/mL) and ARE (50 µg/mL) plus DOX (10µg/mL). (**B**) The quantified cell viability of the DOX-treated 4T1 breast cancer cells with ARE treatment was significantly decreased compared to that of those without ARE treatment. Data are shown as the mean ± SEM (N = 3 for each group, ** *p* < 0.01, one-way ANOVA followed by the Student–Newman–Keuls multiple comparisons post hoc test). Scale bar = 100 μm.

Through immunofluorescence, our results showed that the 4T1 breast cancer cells treated with DOX treatment demonstrated a somewhat damaged morphology of the F-actin structures (Figure 3A). However, the difference in the damaged morphology of the F-actin structures between the ARE treatment group and the Sham group was not noticeable. We further observed that ARE treatment enhanced the damage in the DOX-treated 4T1 breast cancer cells (Figure 3A, ARE+DOX vs. DOX). The most apparent damage was observed in the 4T1 breast cancer cells treated with 50 µg/mL ARE plus DOX at a 10µg/mL concentration, where most F-actin structures were destroyed.

We further quantified the cell viability of the 4T1 breast cancer cells with the Sham, ARE, DOX, and ARE+DOX treatments using the MTT assay. As shown from Figure 3B, our results showed that the cell viability of the 4T1 breast cancer cells treated with ARE treatment showed no significant difference from that of the 4T1 breast cancer cells treated with the Sham treatment (Figure 3B, Sham vs. ARE, *p* > 0.05). Furthermore, the cell viability of the 4T1 breast cancer cells treated with the DOX and ARE+DOX treatments was significantly decreased compared to that of the 4T1 breast cancer cells treated with the Sham treatment (Figure 3B, Sham vs. DOX and ARE+DOX, *p* < 0.01). Our results showed that the cell viability of the 4T1 breast cancer cells was reduced by 5% for the 50 µg/mL ARE treatment, by 25% for the 10µg/mL DOX treatment, and by 60% for the 50 µg/mL ARE+ 10µg/mL DOX treatment. The degree of 4T1 breast cancer cell damage under the 50 µg/mL ARE + 10µg/mL DOX treatment was much larger than the sum of the damage caused by the individual treatments of 50 µg/mL ARE and 10µg/mL DOX. Thus, we suggest that a combination of ARE and DOX treatments can potentially provide synergistic damage in 4T1 breast cancer cells.

### 3.3. ARE Treatment Enhances ROS but Decreases MMP in DOX-Treated 4T1 Breast Cancer Cells

**Figure 4:**
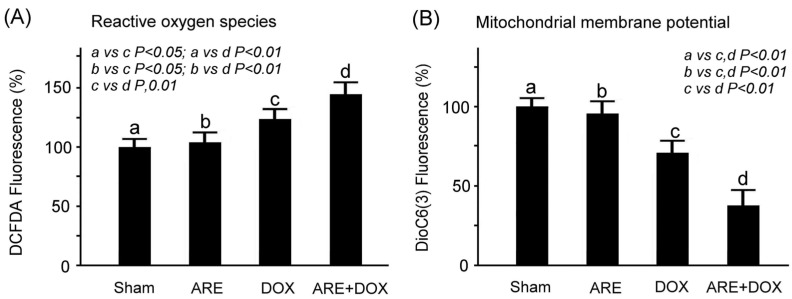
Effect of ARE treatment on ROS and MMP in 4T1 breast cancer cells with DOX chemotherapy: (**A**) The reactive oxygen species and (**B**) mitochondrial membrane potential of the 4T1 breast cancer cells were separately determined via DCFDA and DioC6(3) fluorescence. The 4T1 breast cancer cells treated with DOX (10 µg/mL) had enhanced ROS but reduced MMP, and the AREs further enhanced ROS but decreased MMP in the DOX-treated 4T1 breast cancer cells. The difference in ROS and MMP between the ARE treatment group and the Sham group was not obvious. a: sham, b: ARE, c: DOX, d: ARE+DOX. Data are shown as the mean ± SEM (N = 3 for each group, one-way ANOVA followed by the Student–Newman–Keuls multiple comparisons post hoc test).

Through immunofluorescence, our results showed that DOX treatment enhanced ROS but decreased MMP in the 4T1 breast cancer cells (Figure 4A, Sham vs. DOX, *p* < 0.01; Figure 4B, Sham vs. DOX, *p* < 0.01). There was no significant difference in ROS and MMP between the ARE treatment and Sham groups. Furthermore, AREs enhanced ROS but decreased MMP in the DOX-treated 4T1 breast cancer cells (Figure 4B, ARE+DOX vs. DOX, *p* < 0.01). Enhancing ROS and reducing MPP could accelerate oxidative stress and apoptosis in 4T1 breast cancer cells. Our results revealed that ARE treatment can provide synergistic damage in DOX chemotherapy treatment by enhancing ROS and reducing MPP.

### 3.4. ARE Treatment Alleviates Body Weight Loss and Abnormal Cardiac Function in BALB/c Breast Cancer Mice with DOX Chemotherapy

**Figure 5:**
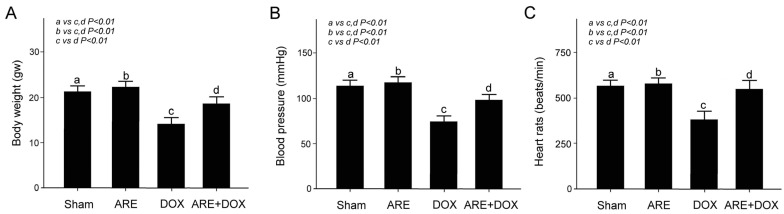
Effect of ARE treatment on body weight and cardiac function in BALB/c breast cancer mice with DOX chemotherapy. Quantified comparison of the body weight (**A**), blood pressure (**B**), and heart rates (**C**) among BALB/c breast cancer mice with Sham, ARE (150 mg/kg), DOX (2.4 mg/kg), and ARE (150 mg/kg) + DOX (2.4 mg/kg) treatments. ARE treatment alleviated body weight loss and abnormal cardiac function induced by DOX chemotherapy in BALB/c breast cancer mice. a: sham, b: ARE, c: DOX, d: ARE+DOX. (N =  8 for each group; values are presented as the mean  ±  SEM, one-way ANOVA followed by the Student–Newman–Keuls multiple comparisons post hoc test).

We quantitatively compared the body weight, blood pressure, and heart rates among the BALB/c breast cancer mice with the Sham, DOX, and ARE+DOX treatments, as shown in Figure 5. There were no significant differences in body weight loss, blood pressure, and heart rate between the ARE treatment and Sham groups in the BALB/c breast cancer mice. The body weight of the BALB/c breast cancer mice after the DOX treatment was significantly reduced compared with that of the mice in the Sham group (Figure 5A, Sham vs. DOX, *p* < 0.01), while the body weight loss of the BALB/c breast cancer mice with DOX treatment was significantly relieved by ARE treatment (Figure 5A, ARE+DOX vs. DOX, *p* < 0.01). Similarly, the blood pressure and heart rates of the BALB/c breast cancer mice after DOX treatment were significantly reduced compared with those of the mice in the Sham group (Figure 5B-C, Sham vs. DOX, *p* < 0.01), while the reduction in the blood pressure and heart rates of the DOX-treated BALB/c breast cancer mice was significantly relieved using ARE treatment (Figure 5B,C, ARE+DOX vs. DOX, *p* < 0.01).

### 3.5. ARE Treatment Alleviates Myocardial Inflammation and Damage in BALB/c Breast Cancer Mice with DOX Chemotherapy

**Figure 6:**
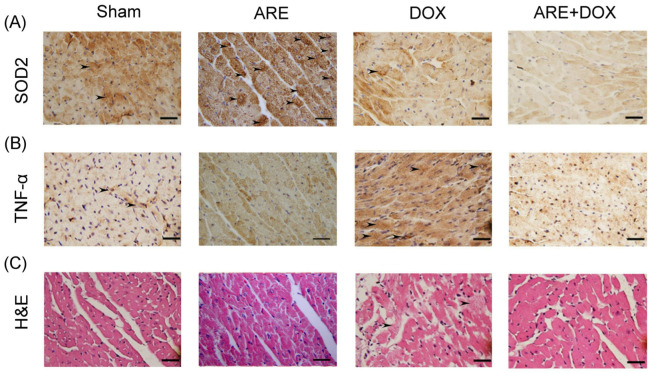
Effect of ARE treatment on myocardial tissue of BALB/c breast cancer mice with DOX chemotherapy: (**A**) Myocardial IHC expression of SOD2 (marked with arrows) among BALB/c breast cancer mice with Sham, ARE (150 mg/kg), DOX (2.4 mg/kg), and ARE (150 mg/kg) + DOX (2.4 mg/kg) treatments. SOD2 expression was obviously reduced after DOX chemotherapy and ARE+DOX treatments. (**B**) Myocardial IHC expression of TNF-α (marked with arrows) in the BALB/c breast cancer mice was obviously enhanced after DOX chemotherapy but obviously reduced after ARE+DOX treatments. (**C**) H&E expression showed myocardial tissue damage (marked with arrows) in the BALB/c breast cancer mice after DOX chemotherapy; however, this was not shown in the BALB/c breast cancer mice after ARE+DOX treatments. Scale bar = 30 μm.

We observed that cardiac SOD2 expression was obviously reduced in BALB/c breast cancer mice following DOX treatment (Figure 6A, Sham vs. DOX). SOD2 plays a significant role in the defense against free radicals. Compared with the Sham-treated mice, the BALB/c breast cancer mice with ARE treatments exhibited higher cardiac SOD2 expression levels (Figure 6A, ARE vs. Sham). Compared with the DOX-treated mice, the BALB/c breast cancer mice with ARE+DOX treatments exhibited even lower cardiac SOD2 expression levels (Figure 6A, ARE+DOX vs. DOX). Our results revealed that ARE treatment was unable to relieve cardiac oxidative stress in the DOX-treated mice.

We observed that the cardiac TNF-α expression was obviously enhanced in the BALB/c breast cancer mice following DOX treatment (Figure 6B, Sham vs. DOX). TNF-α is a robust proinflammatory cytokine that plays an important role in the immune system during inflammation. Compared with the DOX-treated mice, the BALB/c breast cancer mice with ARE+DOX treatments exhibited noticeably reduced cardiac TNF-α expression levels (Figure 6B, ARE+DOX vs. DOX). Our results revealed that ARE treatment alleviated myocardial inflammation induced by DOX chemotherapy in the BALB/c breast cancer mice.

Furthermore, we observed that the myocardial damage was enhanced in the BALB/c breast cancer mice following DOX treatment (Figure 6C, Sham vs. DOX). Compared with the DOX-treated mice, the BALB/c breast cancer mice with ARE+DOX treatments exhibited noticeably reduced myocardial damage (Figure 6C, ARE+DOX vs. DOX). Our results showed that ARE treatment alleviated myocardial damage induced by DOX chemotherapy in the BALB/c breast cancer mice.

### 3.6. ARE Treatment Alleviates Tumor Growth and Myelosuppression of BALB/c Breast Cancer Mice with DOX Chemotherapy

**Figure 7:**
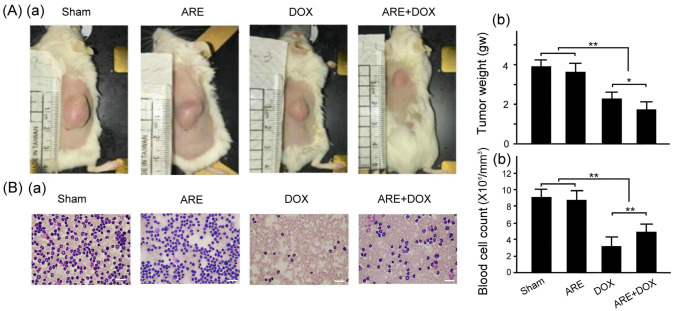
Effect of ARE treatment on tumor growth and myelosuppression in BALB/c breast cancer mice with DOX chemotherapy. Comparisons of (**A**) the tumor size (a) and quantified tumer weight (b); and (**B**) blood cell staining (a) and quantified blood cell count (b) among BALB/c breast cancer mice with the Sham, ARE (150 mg/kg), DOX (2.4 mg/kg), and ARE (150 mg/kg) + DOX (2.4 mg/kg) treatments. ARE treatment alleviated tumor growth and myelosuppression induced by DOX chemotherapy in the BALB/c breast cancer mice (N = 8 for each group; values are presented as the mean  ± SEM, ** *p* < 0.01, * *p* < 0.05, one-way ANOVA followed by the Student–Newman–Keuls multiple comparisons post hoc test). Scale bar = 100 μm.

We measured the tumor weight and blood cell count of the BALB/c breast cancer mice with the ARE and DOX treatments, as shown in Figure 7. The difference in the tumor weight and blood cell count of the BALB/c breast cancer mice between the ARE treatment group and the Sham group was not noticeable. The tumor weight of the BALB/c breast cancer mice after DOX treatment was significantly reduced compared with that of the mice in the Sham group (Figure 7A, Sham vs. DOX, *p* < 0.01), while the tumor weight of the BALB/c breast cancer mice with the DOX treatment was significantly reduced by the ARE treatment (Figure 7A, ARE+DOX vs. DOX, *p* < 0.05). Our results showed that ARE treatment provided an additive inhibition of tumor growth in the BALB/c breast cancer mice with DOX chemotherapy.

We found that the blood cell count of the BALB/c breast cancer mice after DOX treatment was significantly reduced compared with that of the mice in the Sham group (Figure 7B, Sham vs. DOX, *p* < 0.01), while the blood cell count of the BALB/c breast cancer mice with the DOX treatment was significantly increased by the ARE treatment (Figure 7B, ARE+DOX vs. DOX, *p* < 0.01). Therefore, ARE treatment can alleviate the myelosuppression of DOX chemotherapy in BALB/c breast cancer mice.

### 3.7. ARE Treatment Alleviates Immunodeficiency in BALB/c Breast Cancer Mice with DOX Chemotherapy

**Figure 8:**
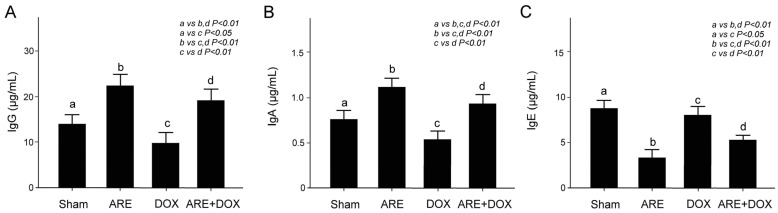
Effect of ARE treatment on immunodeficiency in BALB/c breast cancer mice with DOX chemotherapy. Quantified comparison of blood IgG (**A**), IgA (**B**), and IgE (**C**) levels among BALB/c breast cancer mice with Sham, ARE (150 mg/kg), DOX (2.4 mg/kg), and ARE (150 mg/kg) + DOX (2.4 mg/kg) treatments. ARE treatment enhanced the serum IgG and IgA levels but reduced the IgE level in the DOX chemotherapy of the 4T1 breast cancer cells. a: sham, b: ARE, c: DOX, d: ARE+DOX. (N = 3 for each group; values are presented as the mean ± SEM, one-way ANOVA followed by the Student—Newman–Keuls multiple comparisons post hoc test).

We quantitatively compared the serum IgG, IgA, and IgE levels among the BALB/c breast cancer mice that received the Sham, ARE, DOX, and ARE+DOX treatments, as shown in Figure 8. IgA and IgG play a crucial role in immune function, and IgE plays a crucial role in allergy patients [26]. We found that the IgG and IgA levels of the BALB/c breast cancer mice after ARE treatment were significantly enhanced (Figure 8A,B, Sham vs. ARE, *p* < 0.01), while the IgE levels of the BALB/c breast cancer mice after ARE treatment were significantly reduced (Figure 8C, Sham vs. ARE, *p* < 0.01) compared with those of the mice in the Sham group. The IgG and IgA levels of the BALB/c breast cancer mice after DOX treatment were significantly reduced compared with those of the mice in the Sham group (Figure 8A,B, Sham vs. DOX, *p* < 0.01–0.05), while the IgG and IgA levels of the BALB/c breast cancer mice receiving the DOX treatment were significantly relieved by the ARE treatment (Figure 8A,B, ARE+DOX vs. DOX, *p* < 0.01). There was a noticeable difference in the IgE levels between the BALB/c breast cancer mice after DOX treatment and the mice in the Sham group (Figure 8C, Sham vs. DOX, *p* < 0.05). In contrast, the IgE level of the BALB/c breast cancer mice receiving the DOX treatment was significantly reduced by the ARE treatment (Figure 8C, ARE+DOX vs. DOX, *p* < 0.01). It is worth noting that the ARE treatment significantly enhanced the serum IgG and IgA levels but significantly reduced the IgE levels, in comparison with those of the BALB/c breast cancer mice in both the Sham and DOX treatment groups (Figure 8A–C, Sham vs. DOX vs. ARE+DOX, *p* < 0.01–0.05). Our results show that ARE treatment may boost immunity and relieve allergy symptoms in BALB/c breast cancer mice with DOX chemotherapy.

### 3.8. ARE Treatment Alleviates Osteoporosis in BALB/c Breast Cancer Mice with DOX Chemotherapy

**Figure 9:**
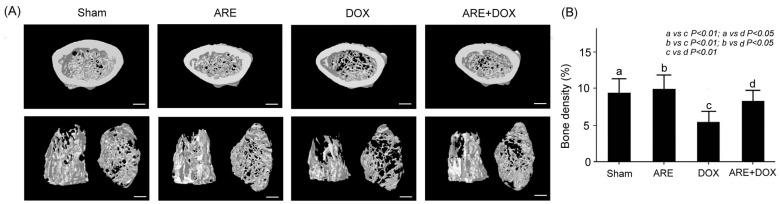
Effect of ARE treatment on osteoporosis in BALB/c breast cancer mice with DOX chemotherapy: (**A**) Cross-sectional imaging of the tibia and trabecular bone among BALB/c breast cancer mice with Sham, ARE (150 mg/kg), DOX (2.4 mg/kg), and ARE (150 mg/kg) + DOX (2.4 mg/kg) treatments. (**B**) Comparison of the quantified bone density of the tibia among BALB/c breast cancer mice with Sham, DOX, and ARE+DOX treatments. ARE treatment alleviated osteoporosis induced by DOX chemotherapy in the BALB/c breast cancer mice. a: sham, b: ARE, c: DOX, d: ARE+DOX. (N = 3 for each group; values are presented as the mean ± SEM, one-way ANOVA followed by the Student–Newman–Keuls multiple comparisons post hoc test). Scale bar = 10 μm.

Through high-resolution microcomputed tomography, we observed that the tibia and trabecular bone densities of the BALB/c breast cancer mice with the Sham, ARE, and ARE+DOX treatments were considerably high; however, those of the BALB/c breast cancer mice with the DOX treatment were low (Figure 9A). The density of the tibia and trabecular bone in the DOX-treated BALB/c breast cancer mice was obviously increased after AR treatment compared to that of the DOX-treated BALB/c breast cancer mice. We quantified the bone density of the BALB/c breast cancer mice with the Sham, ARE, DOX, and ARE+DOX treatments, finding that the bone density of the BALB/c breast cancer mice after the DOX treatment was significantly reduced compared with that of the mice in the Sham group (Figure 9B, Sham vs. DOX, *p* < 0.01); meanwhile, the bone density of the BALB/c breast cancer mice with the DOX treatment was significantly reduced by the ARE treatment (Figure 9B, ARE+DOX vs. DOX, *p* < 0.01). Therefore, ARE treatment can alleviate osteoporosis induced by DOX chemotherapy in BALB/c breast cancer mice.

## 4. Discussion

This study mainly elucidated the mitigating effect of *Anoectochilus roxburghii* extracts (AREs) on the adverse side effects of chemotherapy in mice with breast cancer. The HPLC fingerprints of the AREs indicated that their main bioactive marker compounds were kinsenoside and polysaccharides (Figure 1). In the past, kinsenoside has been proven to have various pharmacological effects, such as hepatoprotection, anti-inflammation, vascular protection, and anti-osteoporosis [17]. Polysaccharides can alleviate liver injury and protect intestinal function by reducing oxidative stress, inflammation, and apoptosis [19]. In addition, polysaccharides have been reported to alleviate impaired intestinal function [19] and can improve high-fat, diet-induced cognitive dysfunction through the gut–brain axis [21].

We observed that the low-dose AREs had a good free radical scavenging ability; however, the free radical scavenging ability gradually decreased with an increase in the dose of the AREs (Figure 2A). In addition, we found that DOX caused chemotherapy-induced damage in the 4T1 breast cancer cells because it reduced cell viability, and ARE treatment further enhanced the DOX-induced cell viability reduction in the 4T1 breast cancer cells (Figure 2B). Although AREs could protect the liver and intestines by reducing oxidative stress, inflammation, and apoptosis [19], we found that ARE treatment may further enhance the DOX chemotherapeutic effect in 4T1 breast cancer cells. Through immunofluorescence, our study revealed that AREs have a synergistic effect with DOX chemotherapy because AREs can further damage 4T1 breast cancer cells under DOX chemotherapy (Figure 3). We found that AREs can enhance reactive oxygen species but reduce the mitochondrial membrane potential in 4T1 breast cancer cells with DOX chemotherapy (Figure 4). These results reveal that DOX chemotherapy may damage 4T1 breast cancer cells via oxidative stress and mitochondrial apoptosis, and that AREs may have an additive damage effect on 4T1 breast cancer cells with DOX chemotherapy by additively enhancing oxidative stress and mitochondrial apoptosis. Figure 2A shows that the AREs under lower-dose treatments had a better antioxidant capacity to scavenge free radical damage than those under higher-dose treatments. Figure 4A shows that the ROS level of the 4T1 breast cancer cells was enhanced with DOX (10 µg/mL). Figure 6A shows that the myocardial SOD2 level increased in the ARE-treated mice but decreased in the ARE + DOX-treated mice. The results suggest that the protective effect of AREs on DOX chemotherapy damage may work through mechanisms other than antioxidative stress. Furthermore, AREs may have an additive damage effect on 4T1 breast cancer cells with DOX chemotherapy by additively enhancing oxidative stress and mitochondrial apoptosis. The additive damage of DOX chemotherapy in 4T1 breast cancer cells has been reported by another study that showed that the extract of *Anoectochilus setaceus* Blume, a close relative Chinese herbal medicine of AR, had strong cytotoxicity against BT474 breast cancer cells with DOX chemotherapy [27].

Among many chemotherapy drugs for human breast cancer, DOX is an effective chemotherapy drug [2]. However, DOX often leads to adverse side effects, such as cardiotoxicity, myelosuppression, immunodeficiency, and osteoporosis [3]. Many cancer patients have symptoms of progressive body fat loss and lean weight, loss of appetite, and anemia, called cachexia [28]. In addition, cachexia may reduce the effectiveness of anticancer chemotherapy and increase chemotherapy toxicity [29]. Skeletal muscle loss is a crucial feature of cancer cachexia. Cancer cachexia is generally believed to be induced by tumor cells producing proinflammatory cytokines, such as interleukin, TNFα, and NF-kB [30]. In addition, cancer causes alterations in protein, lipid, and glucose metabolism, resulting in energy loss and an ineffective utilization of food intake. In 2017, the European Society for Clinical Nutrition and Metabolism defined cachexia as “chronic disease-associated malnutrition with inflammation”, suggesting that cachexia pathology is distinct from starvation and malabsorption, which do not involve inflammation [31]. Through early screening for the risk of cancer cachexia, cancer patients can be administered nutritional interventions before weight loss through supplementation with essential amino acids, omega-3 fatty acids, and Chinese herbal medicines, which may prevent cachexia syndrome [32]. As with cancer patients, BALB/c strain mice were implanted with 4T1 breast cancer cells subcutaneously in the back, and then the changes in the body weight, blood pressure, heart rate, myocardial SOD2 and TNF-α expressions, tumor weight, blood cell count, serum IgG, IgA, and IgE expressions, and bone mineral density were compared among the BALB/c breast cancer mice receiving the Sham, DOX, and ARE+DOX treatments. Our experimental observation found that DOX chemotherapy can cause BALB/c mice to show symptoms similar to cachexia syndromes, such as a loss of appetite and loss of body weight, while AREs can relieve the side effects of body weight loss in BALB/c mice (Figure 5A). In addition, we found that the blood pressure and heart rates of the BALB/c breast cancer mice were significantly reduced after DOX treatment; however, ARE treatment alleviated the reduction in the blood pressure and heart rates of the BALB/c breast cancer mice with DOX treatment (Figure 5B,C). We used H&E and IHC staining on the heart tissue of BALB/c breast cancer mice to evaluate the cardiac injury, as well as the expressions of oxidative stress-related and inflammation-related proteins. We found that DOX chemotherapy can increase the damage to the heart tissue of BALB/c breast cancer mice, as well as reduce the expression of antioxidative stress-related SOD2 and increase the expression of inflammation-related TNF-α.

In this study, we found that the AREs alleviated the damage to the heart tissue of the BALB/c breast cancer mice with DOX chemotherapy and reduced the expression of inflammation-related TNF-α; however, the expression of antioxidative stress-related SOD2 remained unchanged (Figure 6). We found that the AREs further inhibited tumor growth (Figure 7A) but protected against cardiotoxicity and myelosuppression (Figure 7B). These results indicate that the mechanism of action of AREs on normal organ tissue and tumor tissue is obviously different. Similarly, a recent study reported that DOX chemotherapy caused body weight loss and cardiac dysfunction in the 4T1 mouse breast cancer model, while glycyrrhetinic acid treatment significantly attenuated body weight loss and cardiac dysfunction in DOX-treated mice without affecting the antitumor efficacy of DOX chemotherapy [33].

The water extract of *Anoectochilus formosanus* Hayata, a Chinese herbal medicine with close relatives, showed potent tumor-inhibitory activity in BALB/c mice after the subcutaneous transplantation of CT-26 murine colon cancer cells [34]. It was found that the oral administration of the water extract of *A. formosanus* may activate murine immune responses. The same study suggested that the antitumor activity of the extract of *Anoectochilus formosanus* may be associated with its potent immunostimulating effect. We examined the serum of the BALB/c breast cancer mice and found that DOX chemotherapy reduced the serum IgG and IgA levels but did not change the IgE levels; moreover, the AREs enhanced the serum IgG and IgA levels and significantly reduced the IgE levels (Figure 8). IgG and IgA play a crucial role in immune function, and IgE plays a vital role in allergy patients [26]. Our study suggests that AREs are also associated with an immunostimulating effect because they enhance immune function and reduce allergic reactions. We examined microcomputed tomography scans of the tibia and trabecular bone of the BALB/c breast cancer mice and found that ARE treatment attenuated DOX chemotherapy-induced osteoporosis in the BALB/c breast cancer mice (Figure 9). Several reports have shown that the extract of *Anoectochilus formosanus* can ameliorate osteoporosis induced by ovariectomy in rats [35,36]. Kinsenoside, the main active compound of the extract of *Anoectochilus formosanus*, has been reported to prevent ovariectomy-induced bone loss and suppress osteoclastogenesis by regulating classical NF-κB pathways [37]. It is possible that the anti-osteoporotic activity of AREs might be related to their inhibitory effect on osteoclastogenesis. Another possibility is that AREs might reduce DOX chemotherapy-induced osteoporosis in BALB/c breast cancer mice by increasing the intestinal short-chain fatty acid (SCFA) concentration. The prebiotic polysaccharides of AREs have been reported to reduce ovariectomy-induced osteopenia in rats by increasing SCFAs [38].

As observed in our results, we found that the DOX-treated mice developed side effects of chemotherapy after three intraperitoneal injections of DOX. The DOX-treated mice exhibited abnormal body weight loss, a decrease in blood pressure and heart rate, a decrease in appetite and food intake, and a decrease in IgG in the blood. On the contrary, compared to the DOX-treated mice, the ARE+DOX-treated mice showed suppressed weight loss, increased blood pressure and heart rate, increased appetite and food intake, and increased IgG in the blood. Moreover, we found that, compared to the DOX-treated mice, the ARE+DOX-treated mice showed a reduced tumor size. We further considered whether the additional decrease in cancer cell viability after using ARE+DOX is only due to apoptosis or whether necrotic cell death does not occur in this system. Necrosis has traditionally been considered an accidental and genetically unprogrammed form of cell death. Necrosis begins with cell swelling, resulting in cell membrane rupture and the release of cellular cytoplasmic contents into the extracellular space. Unlike tumor-suppressive apoptotic 4T1 breast cancer cell death by DOX chemotherapy, necrosis has been implicated in tumor progression and aggressiveness as a reparative cell death. We found that, compared to the DOX-treated mice, the ARE+DOX-treated mice showed a reduced tumor size. Unfortunately, we did not further examine the apoptotic markers in the tumors. As suggested by Weng et al. [39], AR polysaccharides can exert an antitumor effect by activating the apoptotic protease caspase-3, thereby indirectly or directly promoting the expression of caspase-3 and cell apoptosis. Thus, we suggested that the additional decrease in 4T1 breast cancer cell viability after ARE+DOX treatments was only due to apoptosis, and that necrotic cell death did not occur in this system.

Chung et al. [40] reported an evaluation of the in vitro cytotoxicity and in vivo potential toxicity of AREs. They found that, in an acute oral toxicity study, no mortality or toxicological signs were observed in rats at 1000 or 5000 mg/kg. In a subchronic oral toxicity study, AREs at a dosage of up to 1000 mg/kg produced no mortality or treatment-related adverse effects on general behavior, food intake, body weight, and relative organ weights. No apparent marked changes in the histopathology of the liver and kidney were detected. Data demonstrate that the ARE-induced in vitro cytotoxic effects in cancer cells are associated with a lack of in vivo toxicity. Thus, AREs are suggested to be considered as a potential therapeutic candidate for cancer treatment. In this study, we selected 150 mg/kg AREs to orally treat mice because this dose produced no mortality or treatment-related adverse effects on the mice. According to the results of the MTT assay in Figure 2B, we selected 50 µg/mL AREs to treat the 4T1 breast cancer cells in vitro because it produced the best cytotoxicity for the 4T1 breast cancer cells when doxorubicin was added.

As mentioned earlier, phytochemical investigations have revealed that AR is rich in various functional components, such as kinsenoside and polysaccharides. Kinsenoside has many pharmacological effects, such as liver protection, anti-inflammation, blood vessel protection, and anti-osteoporosis [17]. Furthermore, AR polysaccharides can exert a wide variety of remarkable physiological functions including antidiabetic, hepatoprotective, antioxidant, anti-inflammatory, antitumor, immunomodulatory, sedative, hypnotic, metabolic modulation, anti-aging, and hypolipidemic activities [18,19]. We suggest that AR kinsenoside may play a role in anti-inflammation, blood vessel protection, and anti-osteoporosis, and that AR polysaccharides may play a role in antioxidant, anti-inflammatory, and antitumor activities.

This study proposes the possibility of using AREs to develop therapeutic Chinese herbal medicines for DOX chemotherapy-induced osteoporosis. Since our study mainly explored the relieving effect of AREs on chemotherapy-induced BALB/c breast cancer mice, the BALB/c breast cancer mice were not divided into groups for the experiment, in which they either received the ARK or ARP treatment. We hope that future experiments will individually address the role of the different components of AREs in attenuating chemotherapy toxicity.

## 5. Conclusions

Breast cancer is one of the most common cancers and the leading cause of death in women worldwide, and chemotherapy is the most effective cancer treatment. Our results showed that the DOX treatment obviously reduced body weight, blood pressure, and heart rate and induced myelosuppression, immunodeficiency, myocardial inflammation, and osteoporosis in BALB/c mice with breast cancer, while the ARE pretreatment obviously alleviated the above adverse side effects of chemotherapy in the DOX-treated BALB/c mice with breast cancer. It is worth noting that ARE treatment further reduced the proliferation of breast cancer tumors in the DOX-treated mice. Based on the above results, we suggest that AREs can be used as an adjuvant reliever to chemotherapy because they can alleviate chemotherapy-induced myelosuppression, immunodeficiency, cardiotoxicity, and osteoporosis in BALB/c mice with breast cancer.

## Data Availability

The data are confidential.
